# Spatial Heterogeneity and Influencing Factors of High-Grade Tourist Attractions in the Tibetan Plateau

**DOI:** 10.3390/ijerph20054650

**Published:** 2023-03-06

**Authors:** Shanshan Shi, Mi Li, Ziqiang Li, Jianchao Xi

**Affiliations:** 1 Key Laboratory of Regional Sustainable Development Modeling, Institute of Geographic Sciences and Natural Resources Research, Chinese Academy of Sciences, Beijing 100101, China; 2 University of Chinese Academy of Sciences, Beijing 100049, China; 3 School of Management, Zhengzhou University, Zhengzhou 450001, China

**Keywords:** tourism resources, world tourism destination, spatial distribution characteristics, influence mechanism, sustainable development

## Abstract

The construction of the world tourism destination on the Tibetan Plateau is inseparable from the traditional tourist attractions, which are significant landscape ecological units. Based on the data of high-grade tourist attractions on the Tibetan Plateau, the spatial heterogeneity and influence factors are studied employing the Standard Deviation Ellipse (SDE), Kernel Density Estimation (KDE), spatial autocorrelation (SA), and modified tourism gravity model methods. The results show that: (1) the overall spatial distribution characteristic of high-grade tourist attractions is in the direction of northeast-southwest, with solid centripetal force, and the center of gravity of the ellipse is in Yushu City. (2) The spatial heterogeneity of the kernel density distribution is remarkable, clustered in the southeastern half of the plateau, showing a double nucleus-driven and strip-connected pattern. The distribution among cities has a hierarchical heterogeneity, and the two capital cities of Xining and Lhasa play a crucial role. (3) The high-grade tourist attractions are spatially dependent, with evident characteristics of large dispersion and small clustering, and the spatial association type is mainly negative. (4) This paper verifies the significant single-factor mechanism affecting the spatial distribution from supportive and intrinsic dimensions with natural environmental base, tourism resource endowment, socio-economic development, transportation location constraints, and spatial tourism linkages. Finally, the article provides suggestions for the high-quality development of high-grade tourist attractions on the Tibetan Plateau.

## 1. Introduction

Tourism has become a strategic pillar industry of the Chinese economy, and the spatial pattern of tourism has been dramatically improved [1,2]. A tourist attraction is where tourism and its related activities are one of the main functions that can satisfy the tourists’ needs, such as visiting, leisure, recreation, and fitness, thus contributing to human well-being [3,4]. The grade of tourist attractions symbolizes their construction level and service quality to some extent [5], and the implementation of its classification system has also played a positive role in promoting the development of tourism in China [6]. Recently, the nonprofit Global Wellness Institute (GWI) released “The Global Wellness Economy: Country Rankings”, the first research to measure the wellness economies of 150 nations, with China ranking as the second largest market globally. Additionally, the global wellness tourism market has exceeded 600 billion USD in recent years, with China experiencing the fastest growth and will continue to grow in the post-epidemic era. As the core carrier of tourism economic activities and a substantial resource base for human wellness, it is also a window into the decisive contribution that tourist attractions can bring to regional development. Further, the reasonable spatial distribution of tourism resources is significant for minimizing ineffective investment, increasing tourism activities, and promoting the tourism economy. Therefore, exploring spatial distribution patterns and combination laws of tourist attractions is vital to optimizing regional tourism layout and promoting human wellness development [7].

In academic research, the spatial distribution, agglomeration characteristics, formation mechanisms, and spatial effects of tourist attractions are essential themes [8]. Some scholars have explored tourist attractions in terms of tourism zone division [9], spatial structure [10], the spatial distribution of hotspot areas [11], the evolution of tourist destinations [12], and tourism flow differences [13], accompanied by a refinement of the spatial scale. Others mainly evaluated the current situation and patterns of tourist attractions [14], including the distribution pattern [15], spatial-territorial combination [16], spatial distribution differences [17], spatial-temporal evolution [4,18,19], and “cluster” formation [8]. It was found that the attention to the factors affecting spatial differentiation was mainly focused on topography and geomorphology, resource endowment, policy support, and transportation conditions [20], and the research covered multi-scale spaces such as countries [21], regions [22], provinces [23], and cities [24], etc. The frequently used geological research methods were proximity index [25], kernel density estimation [26], standard deviation ellipse [27], and geographic probes [28]. In addition, along with scientific expeditions, studies on spatial heterogeneity in the Tibetan Plateau have gradually increased. Scholars have explored the spatial distribution of rural tourism villages [29], landscape ecological risks [30], soil moisture [31], and snow depth [32], which have enriched regional research results, but similar studies on the subject of tourism resources have not been conducted. A literature review revealed that research methods on tourist attractions’ spatial distribution and influencing factors were well established, and spatial research types were abundant. However, research on the spatial distribution of tourist attractions in the western region of China is rarely involved, especially the complete geographical unit of the Tibetan Plateau.

The Tibetan Plateau is known as the “roof of the world”, and its formation and evolution have significantly impacted both the natural environment and human activities [33]. Due to the unique characteristics of rich natural resources but a fragile ecological environment and unique culture in an underdeveloped economy, the region also has the critical task of building the foundation of national ecological security, continuously improving the quality of the ecological environment, and promoting high-quality development [34]. In 2010, the Fifth Central Conference on Tibetan Work proposed to build Tibet as “an important world tourism destination”, and President Xi also stressed the need to “build an international ecological tourism destination based on the unique resource endowment of the plateau” during his visit to Qinghai. Additionally, western China faces strategic development opportunities such as rural revitalization, regional tourism, and ecological civilization construction. The efforts to reduce the differences in tourism development between the east and central regions and improve the current imbalance in regional economic development [35] have become necessary. It shows that constructing a world tourism destination has become essential in promoting environmental protection and high-quality development in the study area.

Along with regional economic development, improved transportation conditions, etc., the tourism industry is developing rapidly and becoming increasingly prominent. The total tourism revenue of Qinghai and Tibet reached CNY 60 billion (18.85% of GDP) and CNY 56 billion (31.8% of GDP) in 2019, respectively, which has declined due to the impact of the coronavirus pandemic. Thus, the tourism industry has outstanding performance in promoting sustainable economic development, ecological construction, environmental protection, and tourism destination creation in ecologically fragile areas. As a particular tourism destination with a sizeable spatial scale, scattered core resources, and exceptional organization mode such as the tourism corridor development tandem, high A-grade tourist attractions have become crucial regional tourism distribution centers and tourism network nodes on the Tibetan Plateau. They are also the “hit products” of the region. Accordingly, primary research on high-grade tourist attractions that can promote tourism consumption and meet more people’s aspirations for a better life must be completed urgently [36].

Given the aforementioned, the study adopts geostatistical methods to deeply analyze the spatial distribution characteristics of high-grade tourist attractions and explore the law of spatial heterogeneity. It aims to provide scientific references and suggestions for constructing a world tourism destination and balanced regional tourism development in the Tibetan Plateau.

## 2. Materials and Methods

### 2.1. Study Area

**Figure 1:**
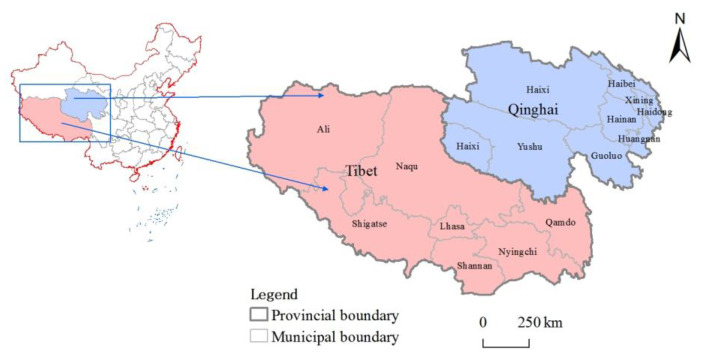
Overview map of the study area (source: GS(2019)1822).

The study area is located in the southwestern part of China’s territory, the central part of which consists of Qinghai Province and the Tibet Autonomous Region (Figure 1). It covers an area of 1,950,700 square kilometers with an average altitude of more than 4000 m and a resident population of less than 10 million, making it a notably sparsely populated region. Among them, Qinghai is located at the intersection of three natural geographic regions in China and one of the multi-ethnic regions. Tibet borders Myanmar, India, Bhutan, and Nepal, forming a natural barrier at the southwestern border of the motherland. The Tibetan Plateau is an essential cultural preserve of Chinese national characteristics and a critical world tourist destination, with rich tourism boutique resources due to its diverse geographical environment and unique ethnic customs, including the World Heritage Sites of Cocoanut Cyril and Potala Palace Complex, national nature reserves such as Mount Everest, Yarlung Tsangpo Grand Canyon, Qinghai Lake, and Sanjiangyuan, with forests, mountains, grasslands, river valleys, and lowlands in the region.

### 2.2. Research Framework

**Figure 2:**
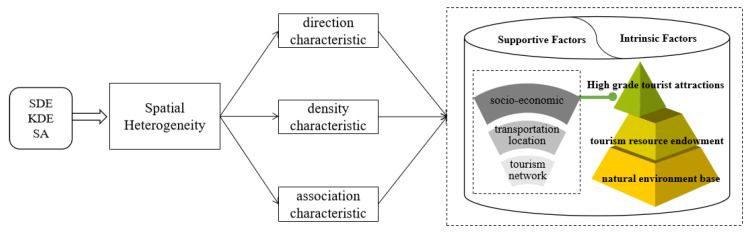
Research framework design.

Based on the push-pull theory, the natural environment base and tourism resources endowment of high-grade scenic spots as the pivot of tourist destinations are the key advantages of the attraction. The higher-quality areas tend to have a greater pull on tourists. On the other hand, high socio-economic levels, good transportation conditions, and frequent inter-city tourism links will drive the supply of high-grade tourist attractions, favorably reflected in the number and quality, the scope of external markets, and the surrounding service support. Thus, referring to the relevant literature [26,36], the single-factor mechanism of action of the spatial heterogeneity distribution of high-grade tourist attractions on the Tibetan plateau was revealed from supportive and intrinsic dimensions (Figure 2).

### 2.3. Research Methods

#### 2.3.1. Standard Deviation Ellipse (SDE)

SDE can reveal the characteristics of geographic elements in two-dimensional space more accurately and quantitatively, including central tendency, spreading, directionality, and density. The method analyzes the development center of gravity and the directional tendency of high-grade tourist attractions on the Tibetan Plateau, mainly from the center of gravity, long axis, and short axis.

#### 2.3.2. Kernel Density Estimation (KDE)

KDE is one of the commonly used methods for geographic hotspot analysis, which has advantages in revealing the distribution characteristics of geographic elements. This paper mainly reflects the spatial distribution characteristics of high-grade tourist attractions on the Tibetan Plateau.

(1) $$\lambda_{h}\left(s\right)=\sum_{i=1}^{n}\;\frac{3}{\pi h^{4}}{\left[1-\frac{{\left(s-s_{i}\right)}^{2}}{h^{2}}\lambda \right]}^{2}$$

where: h denotes the location of the first tourist attraction within the radius space; s denotes the location of the tourist attraction to be estimated; $s_{i}$ denotes the tourist attraction located at the center of s as a circle.

#### 2.3.3. Spatial Autocorrelation (SA)

SA can better measure the potential interdependence between observations within the same distribution area, including global and local autocorrelation. The global autocorrelation is usually expressed by Moran’s I (Equation (2)), which describes the overall distribution of tourist attractions and judges the spatial aggregation characteristics; the local spatial autocorrelation feature is expressed by $I_{i}$ (Equation (3)), which can find the aggregation point by measuring the degree of influence of spatial units on the spatial autocorrelation in the whole study area. The calculation formula is as follows:

(2) $$Moran^{\prime }I=\frac{\sum_{i=1}^{n}\;\sum_{j=1}^{n}\;\left(X_{i}-X\right)\left(X_{j}-X\right)}{S^{2}\sum_{i=1}^{n}\;\sum_{j=1}^{n}\;W_{ij}}$$

(3) $$I_{i}=\frac{X_{i}-X}{S^{2}}\sum_{j=1}^{n}\;\;W_{ij}\left(X_{j}-X\right);S^{2}=\frac{1}{n}\sum_{i=1}^{n}{\left(X_{i}-X\right)}^{2},X=\sum_{i=1}^{n}X_{i}$$

where: $X_{i}$ and $X_{j}$ denotes i and j the number of tourist attractions within the spatial unit; $W_{ij}$ is the spatial weight matrix; n is the number of overall spatial units in the plateau.

#### 2.3.4. Modified Tourism Gravity Model

The tourism gravity model is widely used to study the regional spatial tourism network structure [37]. A modified tourism gravity model was used to measure the structure and interactions of spatial tourism economic linkages between cities in 2021 on the Tibetan Plateau. The calculation equation is as follows:

(4) $$R_{ij}=K_{ij}\frac{\sqrt{P_{i}V_{i}}\cdot \sqrt{P_{j}V_{j}}}{D_{ij}{}^{2}}$$

(5) $$K_{ij}=\frac{V_{i}}{V_{i}+V_{j}};R_{i}=\sum_{j=1}^{n}R_{ij}$$

where: $R_{ij}$ is the tourism linkage intensity of city i and j; $P_{i}$ and $P_{j}$ are the tourism receptions of city i and j; $V_{i}$ and $V_{j}$ are the tourism revenues of city i and j; $d_{ij}$ is the actual shortest road distance between city i and j; considering the difference between tourism linkage and pure economic attraction of the two places, the correction coefficient $K_{ij}$ is calculated by $V_{i}$/($V_{i}$+$V_{j}$) to reflect the weight of city i on the tourism linkage intensity of city j. $R_{i}$ is the total number of tourism economic linkages.

### 2.4. Data Resource

The data on high-grade tourist attractions on the Tibetan Plateau in 2021 were obtained from the official websites of the Department of Culture and Tourism of Qinghai Province and Tibet Autonomous Region. The high-grade tourist attractions referred to in this paper include grades 5A, 4A, and 3A. The spatial coordinate data and the shortest transportation distance data between cities were retrieved through the open platform of Baidu Map (http://lbsyun.baidu.com/, accessed on 2 November 2022). The geographic coordinate data were verified using Gaode Map. Geographic vector data were obtained from the geospatial data cloud (http://www.gscloud.cn/, accessed on 11 March 2022), and socio-economic statistics came from provincial and municipal statistical yearbooks and bulletins.

## 3. Analysis of Results

### 3.1. Spatial Distribution of High-Grade Tourist Attractions on the Tibetan Plateau

#### 3.1.1. Spatial Direction Characteristics

**Figure 3:**
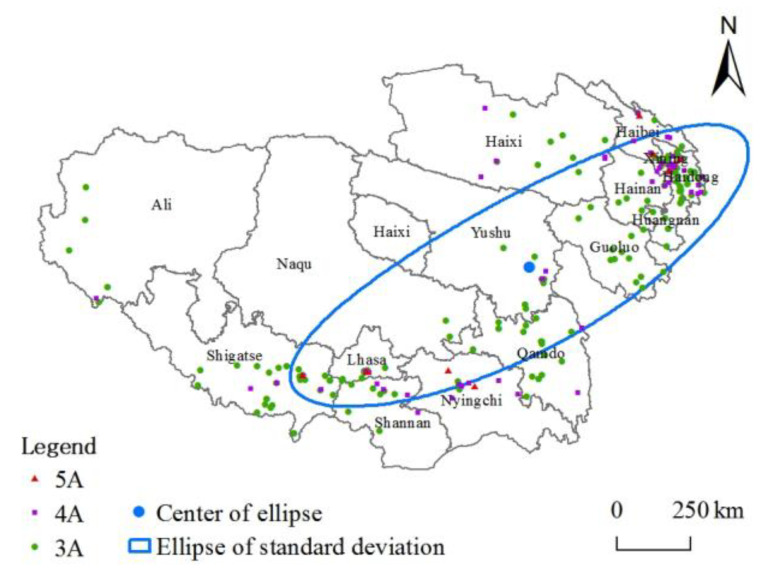
Standard deviation characteristics.

The SDE method can reflect the directional characteristics of the spatial distribution of high-grade tourist attractions on the Tibetan Plateau. The long half-axis of the ellipse (Figure 3) shows that the overall direction of the spatial distribution is “northeast-southwest”, with significant directional characteristics. The shorter half-axis indicates that the data distribution presents a more apparent centripetal force, and the center of gravity of the ellipse is in Yushu City, Yushu Tibetan Autonomous Prefecture.

#### 3.1.2. Spatial Density Characteristics

**Figure 4:**
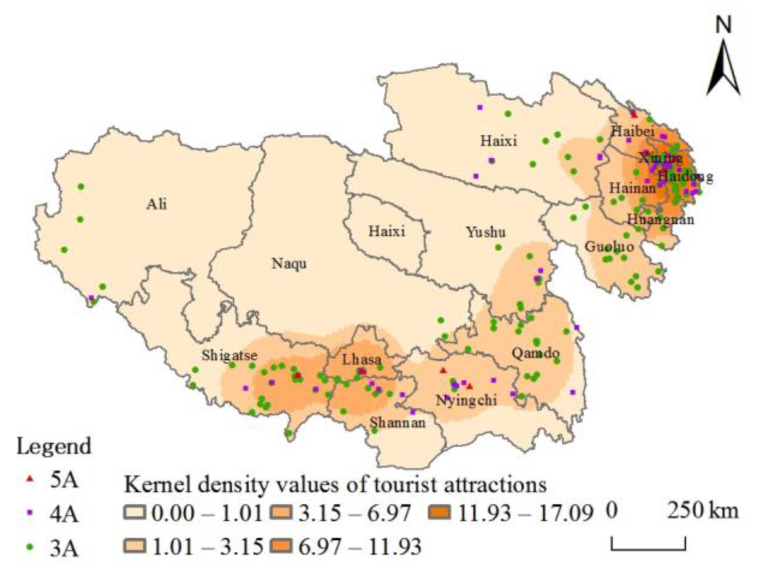
Kernel density characteristics.

Further, the KDE method analyzes the spatial distribution of high-grade tourist attractions on the Tibetan Plateau. As can be seen from Figure 4, the overall kernel density shows the characteristics of “high in the southeast and low in the northwest”, with high-density values concentrated in a few areas and low values in a broader range, forming a spatial pattern of “double core-driven and strip-linked” led by Xining and Lhasa. Among them, the tourist attractions of Xining and surrounding cities have the highest density value and the most prominent radiation influence, forming the infrastructure of a “cluster”. At the same time, Qamdo and Shigatse in Tibet have also developed by relying on the advantages of tourism transportation to promote high-grade tourist attractions. In the “13th Five-Year Plan” period, along with the construction of an all-for-one tourism demonstration area, Qinghai and Tibet strengthened the construction of a tourism corridor and tourism ring road, fine scenic spot project, and the integration of cross-regional tourism was enhanced. The opening of the Lanzhou-Xinjiang Passenger Train and the construction of the Qinghai-Tibet Railway Tourism Belt are conducive to the creation of new tourism growth poles, which gave full play to joint advantages, further driving the construction of tourist attractions in the Tibetan Plateau. It also enhances the connectivity of interactions and promotes the formation of tourism circuits and the development of boutique tourism products.

#### 3.1.3. Spatial Association Characteristics

**Figure 5:**
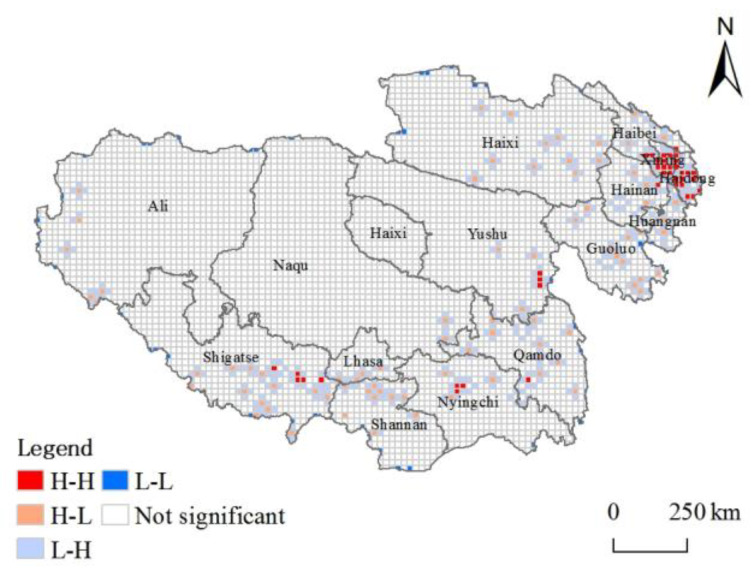
Local autocorrelation of high-grade tourist attractions on the Tibetan Plateau.

Considering the spatial distribution of high-grade tourist attractions on the Tibetan Plateau, the spatial correlation between municipalities may exist. The Moran’s I value was calculated with the help of the spatial autocorrelation statistical tool in ArcGIS 10.8 software. The results showed that global Moran’s I = 0.686 > 0, Z-Score = 2.979 > 1.96, and *p*-Score = 0.003 < 0.05, which passed the significance test, indicating the positive spatial correlation of high-grade tourist attractions on the Tibetan Plateau is strong and spatial clusters are formed. Referring to the existing studies [26] and integrating the geographical and scenic features of the study area, a uniform grid of 20 km × 20 km (Figure 5) was constructed for local autocorrelation analysis, and the spatial distribution of high-grade tourist attractions was divided into four types: H-H, L-H, H-L, and L-L.

Double high type (H-H): indicates that a grid cell within the plateau and its surrounding grid cells in the scenic density value are “double high” area, spatial correlation is strong, mainly distributed in the Hehuang Valley and the “YLN” Region of Tibet, around Xining and Lhasa, to form a local “double core “radiation pattern. The number of grid agglomeration in Xining is the most significant. High-low (H-L): The density value of tourist attractions is high, but the density value of tourist attractions in the surrounding areas is low, showing a polarized pattern of “high in itself and low around” tourism. The spatial grid cells are scattered on a small scale in other areas except for northwest Tibet, and the number is small. Low-high type (L-H): The density value of tourist attractions is low, while the density value of tourist attractions in the surrounding areas is high, which usually belongs to the transition area in the spatial association and has an obvious shadow effect. Double-low type (L-L): It belongs to the “double-low” area with a low-density value for itself and its surrounding tourist attractions. It can be seen from the figure that this type of grid only exists individually near the administrative boundary.

H-H grid cells account for 6.7% of the significant local spatial autocorrelation areas, and H-L and L-H grid cells account for 79.4%. Generally, the spatial correlation of density values of tourist attractions on the Tibetan Plateau varies widely, forming a significant “local agglomeration and overall dispersion” and “center-periphery” structure. The spatial correlation of high-grade tourist attractions is mainly of negative correlation type, and there is still no large-scale clustering distribution [36].

### 3.2. Analysis of the Impact Factors of High-Grade Tourist Attractions on the Tibetan Plateau

The formation and development of tourist attractions are influenced by comprehensive factors, specifically the natural environment base with solid stability and the human geographic elements with strong dynamics. This study focuses on the five aspects of the natural geographical environment, tourism resource endowment, transportation location conditions, socio-economic development, and tourism spatial connection to analyze the factors influencing the spatial distribution of high-grade tourist attractions on the Tibetan Plateau.

#### 3.2.1. Natural Geography

**Figure 6:**
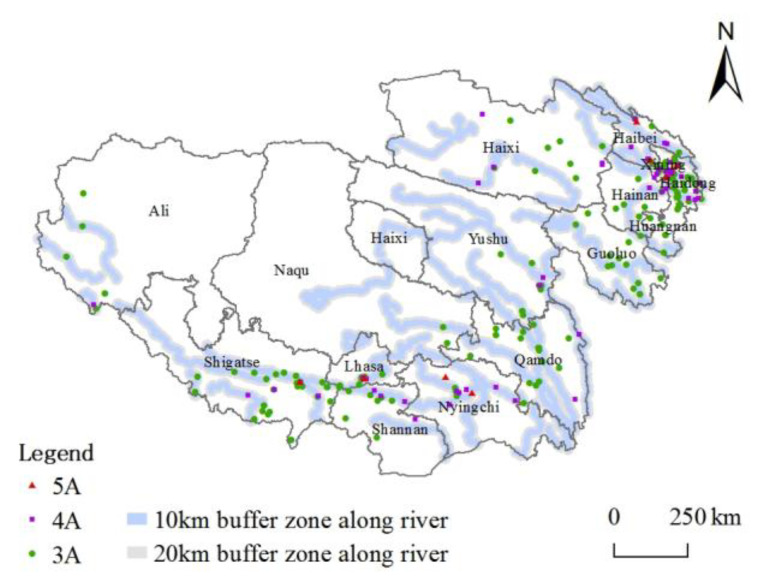
Spatial distribution of tourist attractions and major rivers.

Landscape pattern is a vital landscape component. The topography and distance of water systems can influence the spatial distribution of tourist attractions [38], especially the natural type. Although the Tibetan Plateau is the birthplace of many large rivers, its water resources show significant differences in spatial and temporal distribution [39]. The valley and basin areas rich in water resources and suitable for living are the plateau population towns’ layout points and areas with a high density of tourist attractions and industrial distribution [40]. Further, buffers at different scales of 10 km and 20 km were calculated for the first-, second-, third-, and fourth-grade rivers. The results in Figure 6 show that 173 and 144 high-grade tourist attractions are within 20 km and 10 km buffers from major water systems, respectively, accounting for 70.04% and 58.29% of the total in turn. Overall, the high-grade tourist attractions are mainly distributed within 10–20 km of the water system. They show a noticeable trend of decreasing distance, and the river system substantially constrains the spatial layout of the high-grade tourist attractions in the plateau.

**Figure 7:**
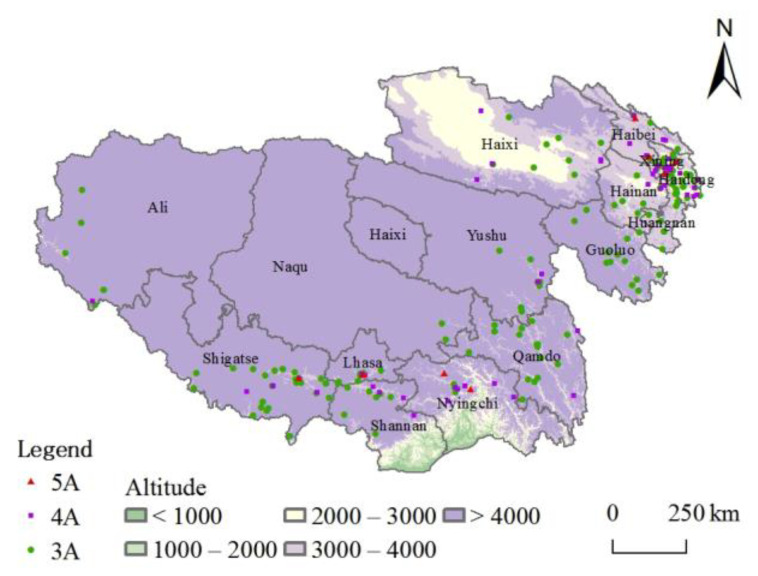
Spatial distribution of tourist attractions and altitude.

As a vast mountain range system, the influence of gravity and external gravity makes the whole plateau’s topography show a trend of inclination from northwest to southeast. The topographic and geomorphological characteristics of different areas can be divided into six sub-regions: the northern Tibetan plateau, the southern Tibetan valley, the Qidam Basin, the Qilian Mountains, the Qinghai Plateau, and the Sichuan-Tibet high mountain valley. The spatial distribution of high-grade tourist attractions on the Tibetan Plateau closely correlates with the topography (Figure 7) and has prominent distribution characteristics along the valleys. The core areas are the Hehuang Valley, the southern Tibetan Valley, and the Qidam Basin. The Qilian Mountains and the Sichuan-Tibet high mountain valley areas also have scattered distribution; in contrast, the Qinghai Plateau and the northern Tibetan Plateau have a minor distribution.

In general, the natural geographic background of the Tibetan Plateau makes the high-grade tourist attractions rely on river systems, and basic lowland distribution features are apparent. Water resources and topography advantages are conducive to the suitability and stability created for developing tourist attractions.

#### 3.2.2. Abundance of Tourism Resources

Tourism resource endowment determines the development potential of tourist destinations. Additionally, it affects the spatial distribution of tourist attractions [2,41,42], and the clustering of tourist attractions strongly depends on tourism resources [8]. The tourism development and construction of the Tibetan Plateau are late. Qinghai and Tibet have achieved zero breakthroughs of grade 5A tourist attractions during the 12th Five-Year Plan. There are 247 grade 3A and above tourist attractions in the region, the number of which is relatively small. Referring to the T/CTAA 0001-2019 “Classification of tourist attraction” group standard issued by the China Tourist Attractions Association, there are 68 natural and 104 humanistic tourist attractions, and the advantages of humanistic resources are more prominent (Table 1). Although the high-grade tourist attractions in Qinghai are ahead of Tibet in terms of development order and scale, the gap between the two places is narrowing.

**Figure 8:**
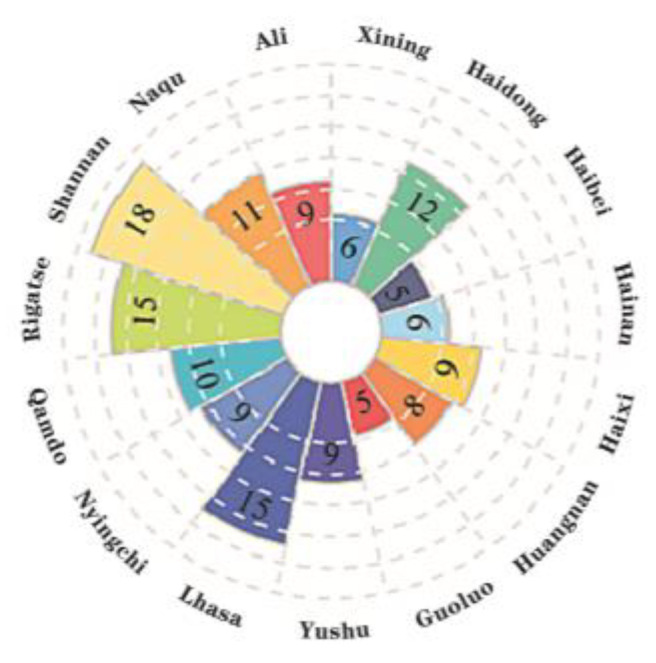
High-quality tourism resources in the cities of the Tibetan Plateau.

World Heritage, National Forest Park, National Geological Park, National Wetland Park, National Nature Reserve, and National Historical and Cultural City are the representatives of regionally distributed high-quality tourism resources, which are essential for creating and developing tourist attractions. Based on the collation of representative high-quality tourism resources (Figure 8), it was found that Xining, Haidong, and Lhasa, have many high-grade tourist attractions distributed and relatively concentrated distribution density, with a high degree of consistency. In addition, although Tibet’s high-quality tourism resources have quantitative advantages compared with Qinghai province, the status quo of high-grade tourist attractions is the opposite, which laterally also indicates that Tibet’s tourism resources have a lower ability to convert to the market. There needs to be more alignment between the development and construction of tourist attractions and the development of the resource background. Matthews et al. [43] pointed out that the superposition of two or more attractions creates a cumulative attraction crucial to attracting tourists, and Ning et al. [8] supported this view. In conclusion, tourism resource endowment may influence the spatial distribution and density characteristics of high-grade tourist attractions on the Tibetan Plateau. The future direction of clustering needs special attention.

#### 3.2.3. Traffic Location Conditions

**Figure 9:**
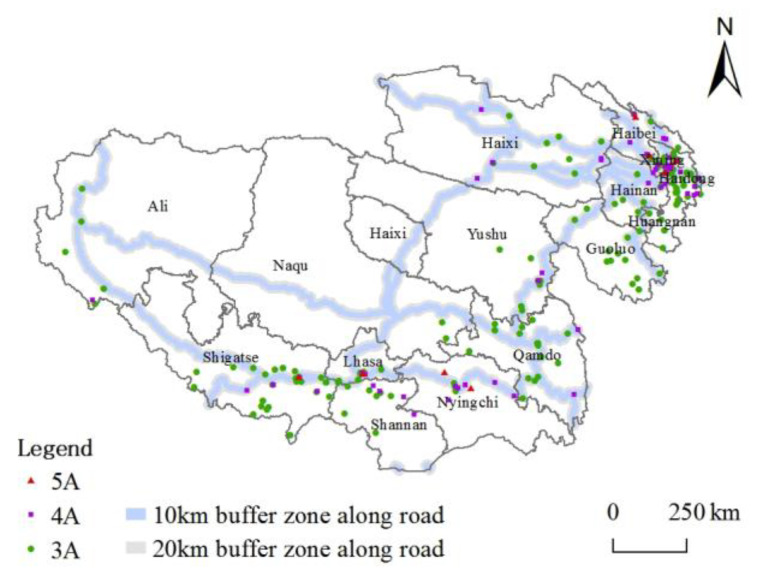
Spatial distribution of tourist attractions and roads.

Traffic accessibility can measure the possibility or potential of tourist attractions to provide services to urban residents and tourists. The good or bad traffic conditions are related to the sustainability of the whole tourism system [44], affecting the effectiveness of stringing together different types of tourist attractions and promoting the optimization of the spatial pattern of high-quality tourist attractions [22]. Xining, Haixi, Haidong, Lhasa, and other transportation hub cities combined with the Qinghai-Tibet, Sichuan-Tibet, and other tourism corridor systems which are interwoven to form a network with high accessibility. The “strip-like” distribution characteristics of high-grade tourist attractions are apparent. With the buffer zone tool, 10 km and 20 km buffer zones are established for the main traffic routes of the railroad, national highways, and tourism corridors on the Tibetan Plateau. Figure 9 shows 139 and 161 high-grade tourist attractions in 10 km and 20 km buffer zones, accounting for 56.28% and 65.18% of the total number, further indicating that traffic development influences the spatial distribution of tourist attractions. From the result, 3A-grade tourist attractions rely most significantly on traffic location. It tends to secure traffic accessibility to expand the range of visitor markets [26], balance the spatial distance, and experience value perception of tourists.

#### 3.2.4. Socio-Economic Development

**Figure 10:**
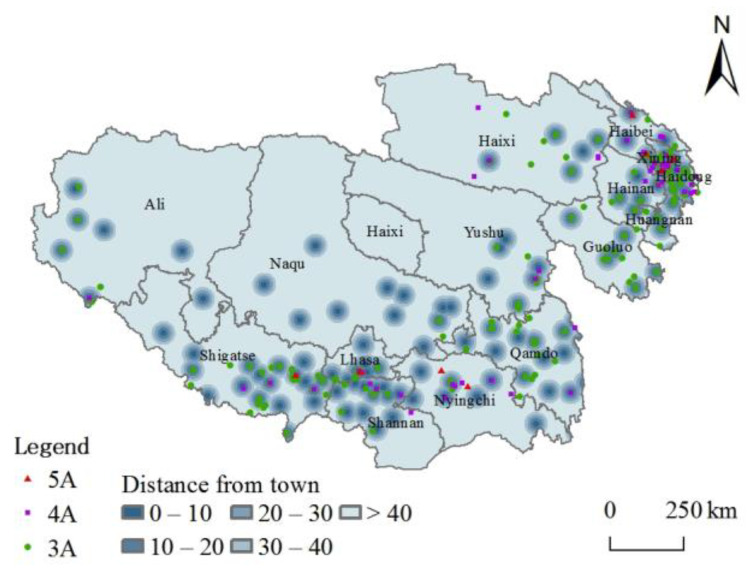
Spatial distribution of tourist attractions and towns.

The high level of socio-economic development promotes the rapid development of tourism and lays the foundation for the formation of high-grade tourist attractions [45]. The complex natural geographical conditions and the unbalanced socio-economic development of various regions also impact the differences. Buffer zones of different scales of 10 km and 20 km were established based on municipal and county-grade administrative centers [26], and information on point-like elements of high-grade tourist attractions within the buffer zones was extracted. The results show (Figure 10) that 143 high-grade tourist attractions are distributed within the 10-km buffer zone of municipal and county administrative centers, accounting for 57.89% of the total, and 187 high-grade tourist attractions are distributed within the 20-km buffer zone, accounting for 75.7% of the total. A better socio-economic development is conducive to the concentration of resources such as population, consumption, information, and investment. The related public services and infrastructure conditions will also be improved. It is found that the overall pattern of high-grade tourist attractions is “dense in the southeast and sparse in the northwest,” with a high degree of spatial consistency with the population [46], towns [47], and rural settlements [48], forming prominent agglomeration centers in Xining, Haidong, and Lhasa. The eastern regions of Tibet and southern Qinghai areas are also developed by the radiation influence of the “Sichuan-Chongqing urban agglomeration”. In contrast, the density of tourist attractions is low in relatively socio-economically backward areas.

#### 3.2.5. Intensity of Tourism Economic Linkages

**Figure 11:**
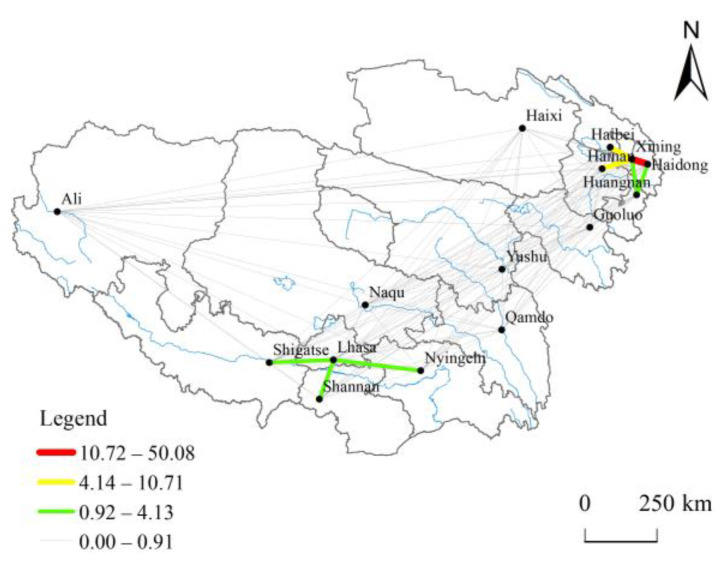
Intensity of tourism economic linkages between cities.

A tourism network is an essential manifestation of high-quality tourism development in terms of spatial layout, which is necessary for tourism destination performance and management. Its structural state also affects tourism’s healthy development [49]. A modified gravity model was used to measure the intensity and total amount of tourism linkages between cities on the Tibetan Plateau. In terms of network structure (Figure 11), there is a divergence in the degree of development of the flow network structure. Cities are connected with different degrees of interaction, forming a parallel pattern of two circles in the northeast and southwest, with Xining and Lhasa as the core. From the perspective of total tourism linkage (Table 2), Xining is the largest, Haidong, Haibei, Hainan, and Lhasa are ranked in descending order, and Ali is the smallest. The long history of tourism development, considerable tourism investment, close tourism economic linkage structure, and frequent factor exchange convergence in Xining and Haidong are conducive to forming the positive development cycle of tourism spatial structure optimization and high-grade tourist attractions agglomeration. At the same time, other regions still need to be enhanced. Overall, there is a specific positive correlation between the intensity of tourism linkage and the spatial distribution of high-grade tourist attractions on the Tibetan plateau. However, the spatial polarization and imbalance characteristics are apparent. The interaction between the eastern and western tourism economic development linkage must be strengthened.

## 4. Discussion

### 4.1. Analysis of the Spatial Distribution and Influence Factors of Tourist Attractions

Studying the definition and classification of tourist attractions is crucial, but attention should also be paid to the spatial patterns and influencing factors in specific regions. In the area of leisure, recreation, and therapeutic recreation, lots of studies have explored the relationship between leisure, well-being, and life satisfaction [50,51,52,53]. Studies also have shown that short-term plateau tourism directly benefits human well-being [54,55]. The high-quality tourism resources, climate, folklore, and high-protein foods of the Tibetan plateau are conducive to human mental relaxation, physical exercise, and therapy [56]. Compared to the related studies, this paper innovatively selects the Tibetan Plateau as an entry point, which enriches the study of spatial heterogeneity of tourism resources in large-scale spatial regions and helps to complement and broaden the research perspective.

Secondly, this study adopts a combination of geographic statistical methods and the modified tourism gravity model to reveal the direction, density, and association characteristics of the spatial distribution of high-grade tourist attractions on the Tibetan Plateau, consistent with existing research paradigms. Similar results have been obtained in different regional studies [17,22,57]. Ma and Yang [5] employed various spatial statistical methods to measure the spatial and temporal distribution characteristics and influencing factors of high-grade tourist attractions in western Hunan. The results found that the regional economic development grade, government policy support, transportation conditions, and tourism resources significantly affect the spatial distribution pattern, and different influencing factors show spatial heterogeneity. Li et al. [26] revealed the spatial distribution characteristics and influencing factors of tourist attractions above grade 3A in the Yellow River basin. Regarding tourism economic linkages, this paper adopted a more refined manner on an inter-municipal basis.

### 4.2. How to Better Promote the Construction of Tourist Attractions in Tibetan Plateau?

Based on the comprehensive analysis of the spatial characteristics and influencing factors of high-grade tourist attractions, we put forward the following scientific suggestions to better guide the rational layout of tourist attractions and promote human well-being.

(1) Developing and constructing tourist attractions should reduce the spatial mismatch with resource endowment. From the time of approval, improve the entry and exit mechanism, focus on “total balance and effective control”, and form a spatial pattern with differences and complementarities coexisting. Increase the quality of existing tourist attractions while appropriately increasing the spatial layout of high A-grade attractions, and accelerate the destination’s competitive advantage through tourism “scenic clusters” [8,58,59]. For grades 4A and 3A, the local government should pay special attention to the construction of facilities and service capacity, and adequately plan the spatial layout of different tourist attractions.
(2) Promote the development of all-for-one tourism, prompting the core attraction of tourism to be more than just sightseeing. Based on the traditional high-grade tourist attractions, the Tibetan Plateau should also promote the strategic reorganization of regional tourism resources and realize the optimal combination of different tourist attractions between cities. Create a core attraction spectrum with diverse structures, provide a variety of tourism product combinations and line services, and focus on the localized environmental atmosphere.
(3) Encourage the development of suitable border tourism. Seize the opportunity of the “Belt and Road” initiative, build world-class tourist attractions that can represent China’s image, and narrow the differences in tourism development between the southeast and northwest regions. In addition, cross-regional tourism exchange should be strengthened to promote the spatial layout of tourist attractions towards the ideal model of “multi-core growth and extended expansion”.
(4) Promoting the construction of the Tangfan Ancient Road, Tibetan-Qiang-Yi Corridor, Ancient Tea Horse Road, and Silk Road is conducive to playing the “point-axis” advantage and promoting the coordinated development of the region. The role of tourism in guiding the development of neighboring towns should be explored to realize the new town development structure of the plateau guided by tourism.

### 4.3. Limitations and Future Research Directions

Due to the limitation of data, it mainly reveals the spatial layout characteristics and influencing factors of high-grade tourist attractions on the Tibetan Plateau, which fails to conduct a dynamic evolution analysis for the time being. In the future, we will further update the long-time series data to deepen the exploration research on the evolution of spatiotemporal patterns. Secondly, this study mainly focused on the single-factor mechanism affecting the spatial distribution of tourist attractions; the interaction effects between factors have yet to be verified, which can be explored through geographic probes.

## 5. Conclusions

Based on the data of tourist attractions of grade 3A and above in 2021 on the Tibetan Plateau, the spatial distribution characteristics and influence mechanism were studied employing the SDE, KDE, SA, and the modified tourism gravity model. The following main conclusions are drawn:

(1) High-grade tourist attractions have typical spatial directional characteristics, with a general direction of “northeast-southwest” and solid centripetal force, and the center of gravity of the ellipse is in Yushu City.
(2) The spatial heterogeneity of kernel density distribution is remarkable, with many clustered in the southeast of the plateau, showing a pattern of “double nucleus-driven and striped”. Additionally, there is a hierarchical heterogeneity, with the two capital cities of Xining and Lhasa playing key roles. The deepening of regional integration is conducive to narrowing the gap in the number of high-grade tourist attractions.
(3) The high-grade tourist attractions in the plateau are spatially dependent, with apparent characteristics of “large scattering and small clustering.” The association type is mainly negative.
(4) It is verified that the single-factor mechanism affecting the spatial distribution of high-grade tourist attractions is significant. Factors affecting tourist attractions’ number and degree of agglomeration include the natural environment base, tourism resources endowment, socio-economic development, transportation location constraints, and tourism economic linkages.

## Figures and Tables

**Table 1:** High-grade tourist attractions on the Tibetan Plateau.

Region	Number of High-Grade Tourist Attractions/pc			Classification of Tourist Attractions/pc				
	5A	4A	3A	Natural Landscape	Humanistic Landscape	Comprehen-sive	Rural and Idyllic	Modern Entertainment
Qinghai	4	38	100	35	49	20	22	16
Tibet	5	23	77	33	55	2	9	6

**Table 2:** Intensity of tourism economic linkages.

City	Xin-ing	Hai-dong	Haibei	Hai-nan	Haixi	Huang-nan	Guo-luo	Yu-shu	Lha-sa	Nying-chi	Qam-do	Shigat-se	Shan-nan	Naq-u	Ali	Ri
Xining	0	62.378	7.551	6.741	1.110	4.558	0.071	0.047	0.215	0.043	0.039	0.034	0.017	0.008	0.005	82.817
Hai-dong	54.333	0	1.213	1.333	0.366	1.929	0.023	0.017	0.085	0.017	0.015	0.014	0.007	0.003	0.002	59.356
Haibei	6.931	1.227	0	0.989	0.203	0.216	0.005	0.006	0.029	0.006	0.005	0.005	0.002	0.001	0.001	9.627
Hainan	5.724	1.253	1.044	0	0.420	0.257	0.015	0.014	0.050	0.010	0.010	0.008	0.004	0.002	0.001	8.810
Haixi	1.056	0.357	0.202	0.419	0	0.112	0.009	0.012	0.149	0.027	0.012	0.023	0.011	0.006	0.003	2.400
Huang-nan	4.256	1.592	0.214	0.267	0.114	0	0.017	0.006	0.033	0.007	0.005	0.005	0.003	0.001	0.001	6.521
Guoluo	0.070	0.023	0.005	0.014	0.020	0.017	0	0.002	0.005	0.002	0.001	0.001	0.000	0.000	0.000	0.160
Yushu	0.046	0.017	0.006	0.014	0.022	0.006	0.002	0	0.021	0.008	0.013	0.003	0.002	0.001	0.000	0.160
Lhasa	0.214	0.085	0.028	0.051	0.151	0.033	0.004	0.021	0	1.918	0.071	2.505	1.211	0.226	0.023	6.542
Nying-chi	0.043	0.017	0.006	0.010	0.028	0.007	0.002	0.008	1.910	0	0.045	0.124	0.066	0.014	0.004	2.282
Qamdo	0.038	0.015	0.005	0.010	0.017	0.005	0.001	0.013	0.070	0.044	0	0.010	0.005	0.004	0.001	0.238
Shigatse	0.034	0.013	0.004	0.008	0.023	0.005	0.001	0.003	2.512	0.124	0.010	0	0.207	0.020	0.006	2.971
Shan-nan	0.017	0.007	0.002	0.004	0.011	0.003	0.000	0.001	1.215	0.066	0.005	0.206	0	0.007	0.002	1.547
Naqu	0.008	0.003	0.001	0.002	0.006	0.001	0.000	0.001	0.216	0.014	0.004	0.020	0.007	0	0.001	0.285
Ali	0.005	0.002	0.001	0.001	0.003	0.001	0.000	0.000	0.023	0.004	0.001	0.006	0.002	0.001	0	0.048

## Data Availability

Not applicable.
